# Optical frequency comb integration in radio telescopes: advancing signal generation and phase calibration

**DOI:** 10.1038/s41377-025-02056-w

**Published:** 2026-01-04

**Authors:** Minji Hyun, Changmin Ahn, Junyong Choi, Jihoon Baek, Woosong Jeong, Do-Heung Je, Do-Young Byun, Jan Wagner, Myoung-Sun Heo, Taehyun Jung, Jungwon Kim

**Affiliations:** 1 Korea Advanced Institute of Science and Technology (KAIST), Daejeon, Korea; 2 Korea Research Institute of Standards and Science (KRISS), Daejeon, Korea; 3 Korea Astronomy and Space Science Institute (KASI), Daejeon, Korea; 4 University of Science and Technology (UST), Daejeon, Korea; 5 Max-Planck Institute for Radio Astronomy, Bonn, Germany

**Keywords:** Frequency combs, Microwave photonics, Astronomical optics

## Abstract

Very long baseline interferometry (VLBI) enables high-angular-resolution observations in astronomy and geodesy by synthesizing a virtual telescope with baselines spanning hundreds to thousands of kilometres. Achieving high instrumental phase stability in VLBI relies on the generation of high-quality, atomic-referenced RF local oscillator (LO) and RF-comb signals for the effective downconversion of celestial RF signals and precise phase calibration, respectively. As observing frequencies move into higher ranges with wider bandwidths, conventional electronic methods face significant challenges in maintaining the quality of these signals. Here, we demonstrate that an optical frequency comb (OFC) can be used as a versatile tool to generate and distribute low-noise and atomic-referenced RF-comb and RF-LO signals in the VLBI telescope. Hydrogen maser-stabilized optical pulses are transmitted over a timing-stabilized fibre link from the observatory building to the VLBI receiver system at the telescope, where photodetection converts them into the required RF signals. In VLBI test observation, we successfully detected VLBI fringes and extracted the RF-combs characteristics in a format suitable for VLBI instrumental phase calibration. These results highlight the high potential of OFC-based technology for enhancing next-generation broadband VLBI measurements, advancing astrophysical research and facilitating intercontinental clock comparison.

## Abstract (web summary)

Optical frequency combs enable simultaneous generation of low-noise phase calibration signals and RF local oscillators in radio astronomy receivers, improving stability and calibration with potential for future higher-frequency observations.

## Graphical abstract

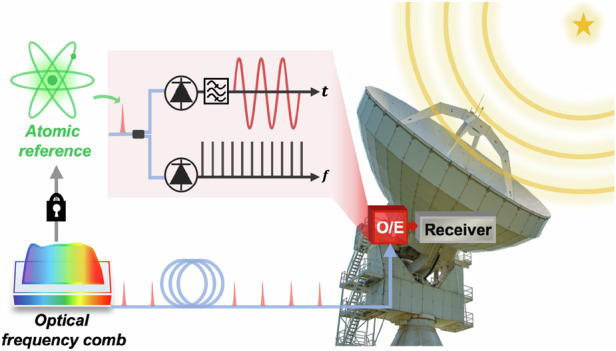

## Introduction

Very Long Baseline Interferometry (VLBI) is a unique measurement technique designed for high angular resolution observations in astronomy and geodesy, which relies on the simultaneous observation of celestial radio sources using distant antennas synchronized to a local atomic clock^1^. By offering an Earth-size virtual telescope that can provide a few tens of micro-arcsecond angular resolution, the VLBI has the potential to, among many other applications, reconstruct the shadows of supermassive black holes^2^. The VLBI measures the time delay and spatial coherence of radio frequency (RF) signals of radio sources reaching multiple antennas. These contain not only geometric delay but also other contributions such as atmospheric effects, on-site instrumental effects, and reference clocks.

While higher frequency VLBI observations enable higher angular resolutions, calibrating the fast fluctuations of water vapour in the troposphere in order to maintain phase coherence of the radio signal coming from the radio source to the antennas becomes more difficult. The best solution to correct for this tropospheric effect is simultaneous observations at multiple frequencies. This allows for effective removal of tropospheric delay errors at higher observing frequencies using the measured delays at the lower frequency^3–5^. Furthermore, aligned multi-frequency VLBI observations are a crucial method for studying the physics of black holes and relativistic jets in active galactic nuclei^6,7^, stellar evolution^8,9^ and geodesy at mm-wavelengths^10^.

To fully exploit the superior performance of astronomic and geodetic VLBI at high frequencies, the instrumental phase drifts at each receiver and signal chain must be precisely calibrated. Thus, a phase calibration (PCAL) signal, i.e., a short pulse train corresponding to low-intensity equidistant frequency tones, is injected to provide spectral lines to each receiver band. The extraction of the phase of these tones in the data processing allows for precise measurement of instrumental phase drift and variation throughout the signal chain of a radio telescope during an observation. However, electronics-based PCAL signal generation has a limited frequency range up to 50 GHz without up-converters^11^, and it requires complicated equalization due to the steep slope in amplitude. This can increase the phase uncertainty of PCAL at higher frequency bands due to a weak PCAL signal or destroy the radio signal from a radio source in the sky due to the high amplitude of the PCAL signal at lower frequency bands. The complexities escalate with wideband receivers, of which broad coverage necessitates intricate calibration and equalization techniques. This challenge becomes particularly critical in sub-millimetre VLBI, where recent studies have shown that instrumental phase calibration is a major limiting factor^12^.

On the other hand, at VLBI radio telescope receivers, low-noise RF local oscillators (LOs) are used to downconvert the signals coming from the sky. Achieving precise measurements demands the use of highly stable RF LOs that follow the stability of the reference clock (frequency standard) at each observatory. As the observation frequency becomes higher for higher resolutions, the generation of higher frequency LO signals with lower phase noise has also become more important. However, the generation of high-frequency microwave signals from the current standard, a hydrogen maser (H-maser), operating at 5 MHz or 10 MHz, mandates the implementation of an intricate multiplication chain, often leading to a compromise in spectral purity. Alternative approaches have been demonstrated using on-site gigahertz-level electronic oscillators and phase-locked loops (PLLs)^13,14^. However, these approaches can only generate a single frequency component at a time, resulting in complexity and bulkiness when applied to multi-frequency receivers demanding multiple LO signals.

Recently, the use of optical frequency comb (OFC) technology in radio telescopes has attracted growing interest in the radio astronomy community. This interest is reflected in several recent efforts, including the deployment of OFC-based distribution systems at the Geodetic Observatory Wettzell^15,16^ and ESA’s deep space stations^17^. Furthermore, OFC-based systems are being actively considered in future space VLBI missions such as the Black Hole Explorer (BHEX)^18–20^.

Here we demonstrate that OFC technology, when implemented directly within VLBI telescopes, provides a simple and high-performance solution to the long-standing challenge of instrumental calibration by enabling the simultaneous generation and distribution of low-noise, atomic-reference-locked PCAL and RF LO signals. Mode-locked lasers (MLLs) and OFC sources can generate ultralow-noise optical pulse trains with quantum-limited timing jitter and repetition-rate phase noise^21,22^. With high-speed photodetection, one can generate low-noise photocurrent pulse trains^23^ that correspond to the RF comb signals up to the photodetector bandwidth and can be directly utilized for the PCAL signals in VLBI telescopes. At the same time, with subsequent bandpass filtering of this photocurrent pulse train, multiple ultralow-noise single-tone RF signals can also be extracted concurrently, which can then be used for VLBI LO signals.

In this work, optical pulse trains locked to the H-maser are delivered from the observatory building to the VLBI antenna via a ~100 m-long timing-stabilized fibre link. At the VLBI antenna, a broadband RF-comb signal with 40 MHz comb spacing is generated by direct photodetection of delivered pulses at sky-frequencies up to 50 GHz, and is further injected into the operating PCAL system of the multi-frequency receiver. In addition, 16.64-GHz and 19.2-GHz LO signals were generated, with a measured phase noise level of -126 dBc/Hz at a 100-kHz offset. The true phase noise is expected to be even lower, as this result is limited by the noise floor of the measurement instrument. Overall, the phase noise and long-term stability of the signals follow the characteristics of the H-maser source. Note that, while there were earlier works on optical link-based time and frequency distribution for radio astronomy, including optical carrier, RF frequency, and frequency comb transfer^24–28^, this is the first demonstration of the transfer of the comb signals directly to the antenna receiver room to generate broadband PCAL and ultralow-noise RF LO signals, to the best of our knowledge. In our first experiment, a successful fringe detection and extraction of PCAL signals at 22 GHz was obtained. Furthermore, as the optical frequency comb operates as a coherent link between optical and microwave frequencies, our approach holds the potential to enhance precision timing in VLBI applications by serving as the last-mile delivery of next-generation optical clocks to the VLBI system, which is critical for intercontinental clock comparison^29,30^. Note that the stability of optical atomic clocks can be distributed over fibre links by transferring a CW laser stabilized to the clock. In this context, our system serves as a crucial interface that converts the delivered optical stability into comb-based PCAL and LO signals directly at the VLBI receiver.

## Results

### Concept and experimental setup

**Fig. 1:**
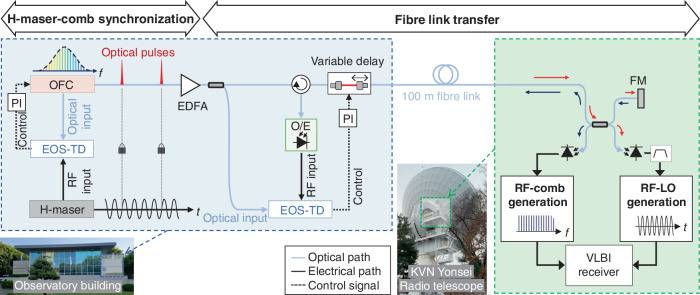
Photonic generation and distribution of RF signals at the VLBI radio telescope. Schematic of the demonstrated photonic system that delivers H-maser-locked optical pulses from the observatory building to the antenna room to generate RF-LO and RF-comb signals for downconversion and phase calibration at the VLBI receiver. EDFA Erbium-doped fibre amplifier, EOS-TD electro-optic sampling-based timing detector, FM Faraday-rotating mirror, OFC optical frequency comb (40-MHz mode-locked Er-fibre laser comb), O/E optical-to-electrical conversion, PI proportional-integral servo controller

Figure 1 illustrates the conceptual diagram of the OFC-based system for generating the PCAL and LO signals at the Korean VLBI Network (KVN) Yonsei radio telescope in Seoul, South Korea. An OFC source (40-MHz repetition-rate mode-locked Er-fibre laser comb) and an H-maser (Kvarz CH1-75A) are placed at the observatory building (H-maser room), while the RF signals generation is conducted in the receiver room at the antenna, denoted by the dashed box in the telescope image (on the right side of Fig. 1). The optical pulse train is transmitted through a ~100-m-long single-mode fibre link to the antenna. At the end of the link, an optical fibre coupler splits the optical pulses into two branches: one is directed toward a Faraday-rotating mirror (FM in Fig. 1) that reflects the signal toward the H-maser room for the link stabilization, while the other branch is used for the RF signals generation for the radio telescope.

The OFC (mode-locked fibre laser) is synchronized with the local atomic clock, the H-maser, as depicted in Fig. S1 in Supplementary Information. The repetition rate of the comb laser is set to 40 MHz based on the requirements of the KVN PCAL system. An 800-MHz microwave signal is generated through frequency multiplication of an oven-controlled crystal oscillator (OCXO) phase-locked to the H-maser. The phase difference between this microwave signal and the optical pulse is detected by an electro-optic sampling-based timing detector (EOS-TD)^31–33^. The EOS-TD is a phase-biased Sagnac-loop fibre interferometer that can convert the timing error between the optical pulses and the electric signals (such as sinusoidal RF signals or periodic electric pulse train) into the intensity change of the interferometer outputs (see Fig. S2 in Supplementary Information). After detecting the Sagnac-loop outputs using a balanced photodetector (BPD), an error signal proportional to the timing error between the optical pulse and the electric signal can be obtained with sub-fs resolution and drift^33^. The voltage output from the EOS-TD is fed into a proportional-integral (PI) controller, amplified by a high-gain voltage amplifier, and used to control the piezo actuator that adjusts the cavity length of the mode-locked fibre laser. These optical pulses, which deliver the stability of the H-maser, are transferred through a fibre link to the radio telescope antenna for PCAL signal generation and LO extraction (see Fig. S3 in Supplementary Information) for the multi-band VLBI receiver.

We stabilized the group delay of the ~100-metre-long optical fibre link between the H-maser room and the antenna room to ensure precise timing signal delivery. The timing jitter of the optical pulse train directly determines the phase noise of the repetition rate and its harmonics in the extracted microwave signals. Since the optical pulses propagate at the group velocity in the fibre, fluctuations in group delay, caused by environmental factors such as mechanical stress, acoustic noise and temperature loads, directly result in timing jitter at the receiver. Therefore, minimizing group delay fluctuations is essential for preserving the timing stability of the transmitted signal. To compensate for these delay variations, another EOS-TD is employed to measure and compensate for the fibre delay error (see Fig. S4a in Supplementary Information). Compared to the well-established optical cross-correlation technique^34–37^, the main advantages of using the EOS-TD for link stabilization is that it can detect the timing error between the optical pulses and the electric signals with sub-fs resolution even when the optical pulsewidth is much longer than ps^38^, therefore it does not require strict dispersion compensation in the optical pulse distribution. The use of electric signals for measuring the optical pulse position also allows for a much wider timing detection range of more than tens to hundreds of ps.

After amplification, optical pulses from the mode-locked fibre laser comb are split into two streams: one used as a reference signal for the EOS-TD and the other sent through a dispersion-compensating fibre and a variable delay element for length compensation before transmission to the antenna. The reflected pulses are converted into an electronic signal and applied to the EOS-TD. Two RF signals – either harmonics from photocurrent pulses or a 1.4 GHz microwave signal (Fig. S4b) – can be used for timing stabilization, with feedback provided by a PI controller and a high-gain voltage amplifier to the variable delay for accurate fibre link timing.

Through the timing-stabilized fibre link, optical pulses are converted to low-jitter photocurrent pulses via a high-linearity photodiode at the antenna, serving as a narrowband PCAL signal for the VLBI system with low optical power (300 μW) to prevent interference with astronomical signals, while still high enough to extract the PCAL signal with a sufficient signal-to-noise ratio. The same photocurrent pulses are used to generate a low-phase-noise microwave signal for the LO, where pulsed illumination provides a lower noise floor and higher power efficiency. To boost the microwave signal, a five-stage fibre-based pulse repetition-rate multiplier^39,40^ increases the repetition rate to 1.28 GHz, enhancing power at the 13th harmonic (16.64 GHz) and 15th harmonic (19.2 GHz) for the LO signal, with the harmonics filtered and amplified to meet receiver power requirements. More detailed information on the experiment setup can be found in Methods and Figs. S1-S4 in Supplementary Information.

### H-maser-comb synchronization

**Fig. 2:**
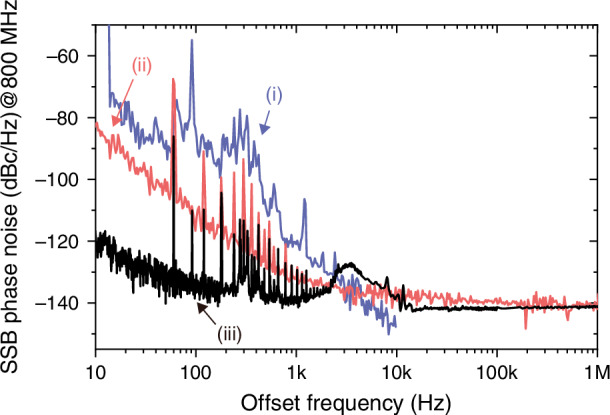
H-maser-comb synchronization performance. (i) Absolute repetition-rate phase noise of the free-running 40-MHz OFC source (scaled to 800-MHz carrier frequency). (ii) Absolute phase noise of the 800 MHz microwave signal from the OCXO phase-locked to the H-maser when measured by the signal source analyser. (iii) Residual phase noise for H-maser-comb synchronization when measured by the out-of-loop EOS-TD

Figure 2 shows the phase noise spectra related to H-maser-comb synchronization. Here, the fibre comb is synchronized to the 800-MHz output of an OCXO, which is phase-locked to the H-maser (see Fig. S1). Curve (i) shows the repetition-rate phase noise of the free-running 40-MHz fibre comb laser before synchronization, when scaled to 800-MHz carrier frequency (20th harmonic). Curve (ii) represents the absolute phase noise of an 800 MHz microwave signal generated from the OCXO phase-locked to the H-maser, measured by the signal source analyser (SSA). The residual phase noise for H-maser-comb synchronization, measured by the out-of-loop EOS-TD, is presented by curve (iii). These results show that the residual phase noise in the H-maser-comb synchronization (curve (iii)) remains sufficiently low compared to the H-maser-locked OCXO noise (curve (ii)), indicating effective phase stability transfer of the H-maser to the antenna room via optical pulse trains.

### Fibre link stabilization

**Fig. 3:**
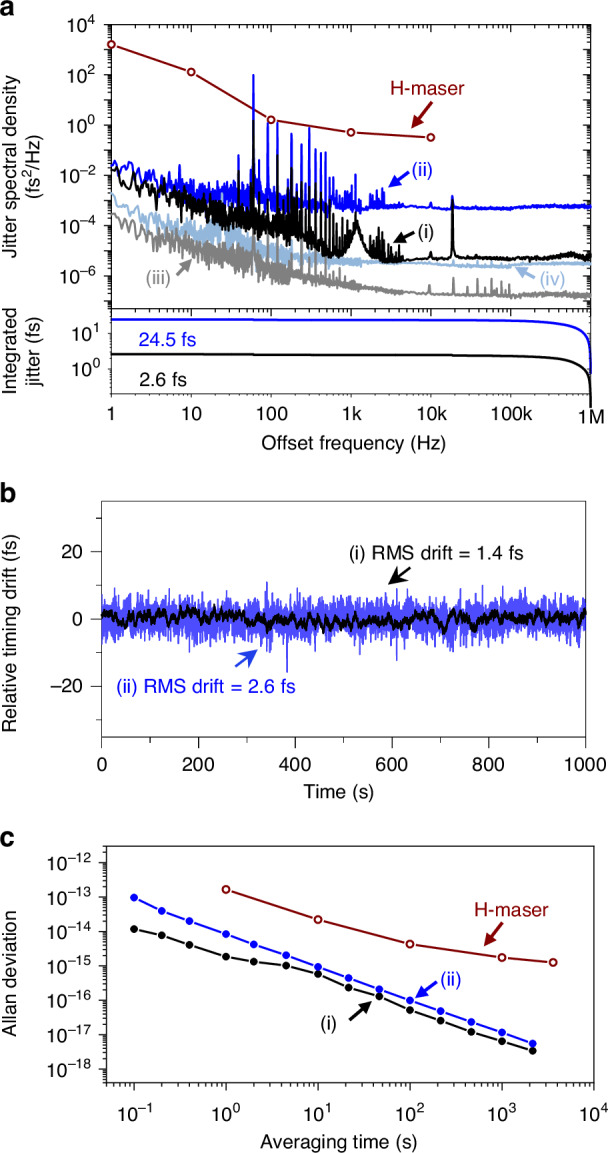
Fibre link stabilization performance. **a** Residual jitter spectral density using (i) photocurrent pulses and (ii) extracted microwave, their measurement backgrounds (iii), (iv), respectively. **b** Long-term residual timing drift. **c** Allan deviation versus averaging time using (i) photocurrent pulses and (ii) extracted microwave. For comparison, H-maser performance from manufacturer’s test sheet is presented

To guarantee complete transfer of H-maser stability, the residual noise of the fibre link should be far lower than that of H-maser. The measured residual timing jitter spectral density of the fibre link stabilization is presented in Fig. 3a. For comparison, the phase noise of the H-maser, provided in the manufacturer’s test sheet, is also included. Curve (i) shows the residual jitter density when the photocurrent pulses are used for the RF input to the EOS-TD. The residual jitter density is 4.6 × 10^-4^ fs^2^/Hz, 2.4 × 10^-5^ fs^2^/Hz, and 7 × 10^-6^ fs^2^/Hz at 10-Hz, 1-kHz, and 1-MHz frequency, respectively. Curve (ii) shows the phase noise when an extracted 1.4-GHz microwave signal is used for the RF input to the EOS-TD. The residual jitter density at 10-Hz, 1-kHz, and 1-MHz offset is 3 × 10^−3^ fs^2^/Hz, 8 × 10^−4^ fs^2^/Hz, and 6 × 10^−4^ fs^2^/Hz, respectively. The integrated rms timing jitters using photocurrent pulses and microwave signal are 2.6 fs and 24.5 fs integrated from 1 Hz to 1 MHz, respectively. Figure 3b shows the long-term residual timing drift measurement for both methods, with the rms timing drifts over 1000 s of 1.4 fs (photocurrent pulse) and 2.6 fs (microwave signal). This fibre link stabilization system can operate over 30,000 s without loss of coherence, exceeding typical VLBI observation scan durations under 1000 s. The computed fractional frequency instability based on this timing drift measurement is shown in Fig. 3c, starting from 1.2 × 10^−14^ (9.6 × 10^−14^) at 0.1 s averaging time and reaches to <6.4 × 10^−18^ ( <1.2 × 10^−^^17^) at 1000 s averaging time for photocurrent pulses (microwave signal). The photocurrent pulse-based stabilization achieves better performance due to higher detection sensitivity and reduced relative jitter, and it simplifies the setup by eliminating RF amplifiers and bandpass filters, thereby reducing background noise. Given that both methods produce residual noise levels significantly below H-maser phase noise, either configuration with EOS-TD is suitable for link stabilization. These results are benchmarked against other state-of-the-art optical frequency comb-based link stabilization techniques (see Fig. S5 of Supplementary Information) and demonstrate comparable or superior stability. Moreover, since the optical frequency comb is synchronized to an H-maser and the residual noise of both the synchronization loop and the fibre link stabilization is well below the H-maser’s intrinsic phase noise and long-term frequency stability (Allan deviation), the phase of the extracted microwave signals preserves the full accuracy of the H-maser reference. For future systems requiring full optical clock dissemination, performance can be further enhanced by increasing the EOS-TD RF input frequency or by adopting balanced optical cross-correlation-based^36^ stabilization.

### RF-comb signal generation

**Fig. 4:**
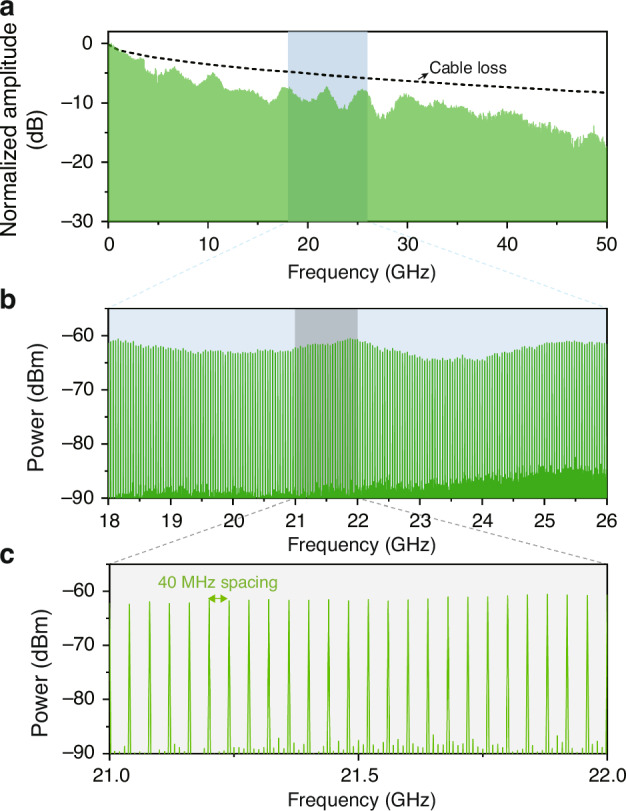
RF-comb signal generation results. **a** Generated RF-comb signal up to 50 GHz presented in normalized amplitude. **b** Power spectrum of RF-comb signal at K-band. **c** Zoom-in of the RF-comb power spectrum showing 40-MHz spacing. The resolution bandwidth is set to 51 kHz

The RF spectrum of the RF-comb signals generated in the antenna room was measured using a 50-GHz bandwidth RF spectrum analyser (Keysight, N9030A) with a resolution bandwidth of 51 kHz. It contains integer harmonics of repetition rate within the photodiode bandwidth, shown as normalized amplitude in Fig. 4a. The used RF cable (Gore, Phaseflex) has frequency-dependent insertion loss as plotted by a dashed line in Fig. 4a, indicating that the RF-comb amplitude remains nearly uniform up to 50 GHz once the cable loss is accounted for. Note that the frequency range of RF-comb can be extended beyond 100 GHz with the higher bandwidth photodiodes^41^, which would not be accomplished by conventional electrical RF-comb generators. The power spectrum of the RF-comb signal at K-band injected to the VLBI receiver is shown in Fig. 4b. Each frequency component is equal-distanced by the repetition rate of the optical pulse, 40 MHz in this case (see Fig. 4c), covering the entire K-band as required for the PCAL signal for the KVN-Yonsei telescope.

### RF-LO signal generation

**Fig. 5:**
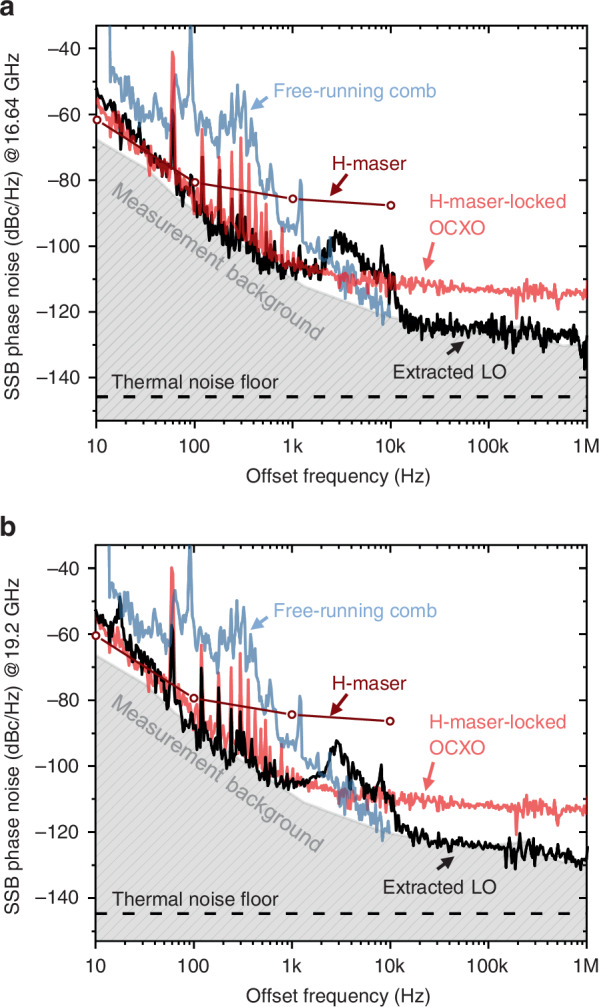
RF-LO signal phase noise performance. Measured single-sideband (SSB) absolute phase noise performances of the extracted LOs at **a** 16.64 GHz and **b** 19.2 GHz. The thermal noise floor is computed from the input optical power to the photodiode and the RF amplifier noise figure

Figure 5 shows the absolute phase noise performance of the extracted RF-LO signals measured in the antenna receiver room by a signal-source analyser (SSA). The broad peak around 3 kHz is the servo bump resulting from the H maser-comb synchronization PLL (curve (iii) in Fig. 2). Free-running comb noise is measured using the same SSA by O-E conversion and extracting the fundamental harmonic of the laser, then rescaled to the carrier frequency of the corresponding LO. Within the locking bandwidth, the phase noise of the 16.64-GHz (19.2-GHz) signal closely follows the phase noise of the H-maser-locked OCXO, indicating a complete transfer of frequency stability. It’s worth noting that the significant spurious noises ranging from 100 Hz to 1 kHz originate from the H-maser system, affecting not only the H-maser-laser stability transfer loop but also the phase noise of the LO itself. The SSB phase noise reaches −126 dBc/Hz (−124 dBc/Hz) at a 100 kHz offset frequency. Note that these results are limited by the instrumental noise of SSA in the offset frequency range of <10 Hz and >200 kHz. Given the input optical power to the photodiode, the thermal noise-limited noise floor^42^ is projected to be −149 dBc/Hz (−148 dBc/Hz) at 16.64 GHz (19.2 GHz), and with the RF amplifier noise figure included, it will reach −146 dBc/Hz (−145 dBc/Hz). These results indicate that the actual RF-LO phase noise is considerably lower than the measured result, which is limited by the SSA instrument noise. For the operation of KVN-Yonsei, a photonic RF-LO signal at 16.64 GHz was injected into the VLBI receiver.

### VLBI observation and PCAL extraction

The first VLBI experiment using the KVN-Yonsei (KY) radio telescope, integrated with photonic-generated PCAL and LO signal chains, was conducted from 21:48 UTC on 14 May 2024 to 02:05 UTC on 15 May 2024. All four KVN radio telescopes – Yonsei (KY), Ulsan (KU), Tamna (KT), and Pyeongchang (KC) – participated, observing 40 well-known VLBI sources in 2-minute scans per source. A simultaneous dual-frequency setup at 22 GHz and 43 GHz was employed with multi-frequency receivers in both right-hand circular polarization (RHCP) and left-hand circular polarization (LHCP), each with a 512-MHz bandwidth (512 MHz × 4 channels), resulting in an 8-Gbps data rate. Photonic PCAL signals were injected into the RHC coupling port of the 22-GHz receiver at KY.

**Fig. 6:**
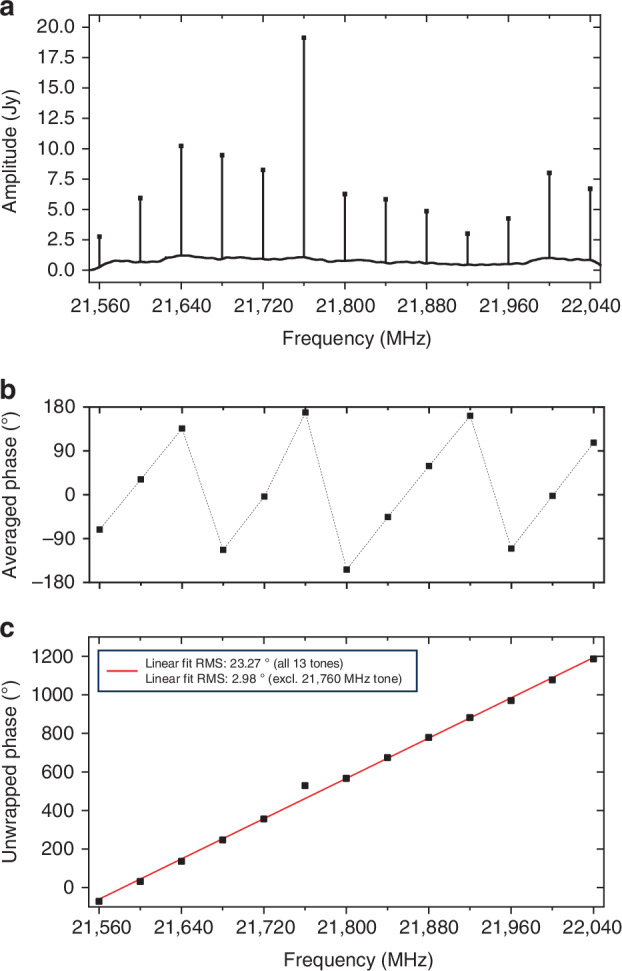
Time-averaged power and phase spectra of PCAL tones. **a** Power spectrum of PCAL tones observed with the KVN Yonsei (KY) radio telescope. Thirteen distinct tones are clearly identified within a 512 MHz bandwidth centred at 21.806 GHz in RHCP. The tones are evenly spaced at 40 MHz intervals. **b** Phase spectrum of PCAL tones. **c** Unwrapped phase spectrum after removing 2π ambiguity. Linear fits are applied to both the full tones and a subset excluding 21,760 MHz tone, with the corresponding RMS residuals of group delay indicated. All spectra are averaged over a duration of 2 minutes

**Fig. 7:**
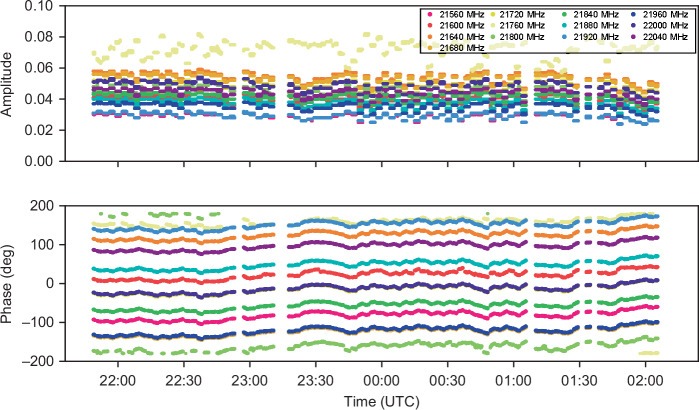
Amplitude and phase of detected PCAL tones during the observation. A total of 13 PCAL tones, spaced by 40 MHz, are shown within a 512 MHz bandwidth centred at 22 GHz (RHCP), as observed with the KVN Yonsei (KY) radio telescope. The frequencies of tones are 21,560, 21,600, 21,640, 21,680, 21,720, 21,760, 21,800, 21,840, 21,880, 21,920, 21,960, 22,000, and 22,040 MHz

The interferometric correlation was processed using the DiFX software correlator^43^ with 1-s integration time and 62.5-kHz frequency sampling. Fringe fitting was performed using the NRAO Astronomical Image Processing System (AIPS), with KU as the reference antenna. The fringe detection at KY showed a signal-to-noise ratio (SNR) ranging from 20 to 350, with an overall detection rate of approximately 92% across frequency and polarization bands, indicating stable performance of the photonic RF-LO throughout the observation. The PCAL tone extraction was performed, confirming stable amplitudes and phases with minimal phase excursions during the observation period (as shown in Figs. 6 and 7).

Both the averaged phase and unwrapped phase across frequency are presented in Fig. 6, clearly illustrating the PCAL phase characteristics. The RMS residuals after linear fitting to the unwrapped phase are also shown for both the full set of tones and a subset excluding the 21,760 MHz tone, which exhibited relatively unstable tone quality. Please note that the instability observed at the 21,760 MHz tone does not originate from the OFC. A potential cause is spectral folding or mixing artifacts arising from overlap between the PCAL tone and strong LO-related components in the IF band. This issue may be mitigated in future observations by slightly shifting the RF observation frequency to avoid such interference. Based on the subset excluding 21,760 MHz tone, the measured group delay of the PCAL measurement is approximately 17 ps at 22 GHz over a 480 MHz span, reflecting the cumulative effects of the entire signal chain from photonic PCAL generation to correlation processing. While the intrinsic jitter of the photonic PCAL itself is expected to be well below 1 ps, this result demonstrates robust system-level operation under real observational conditions. The results in Fig. 7 further demonstrate the robustness of photonic RF signal generation and distribution, validating its effectiveness for VLBI phase calibration and high-frequency observation. A direct performance comparison with and without PCAL calibration will become feasible in future experiments, once a second photonic PCAL unit is installed at another KVN station.

## Discussion

In summary, we have proposed and demonstrated the OFC-based RF signal distribution and generation, offering low-phase noise signals for multi-frequency VLBI receivers. The current demonstration involves transmitting an OFC signal to the antenna, extracting the PCAL and LO signals, and then injecting them into the VLBI receiver. At present, the frequency range of PCAL signal generation has been demonstrated up to 50 GHz, limited by the frequency range of the RF analyser used. The frequency range of the PCAL signal would be determined by the bandwidth of the optical signal and the capabilities of the photodiode. Given the broad bandwidth of optical signals, the bandwidth of the photodiode would set the upper limits, with commercial parts exceeding 100 GHz readily available. Therefore, expanding the frequency range of the proposed system for mm-wavelength VLBI applications is feasible. For example, the KVN-style multi-frequency receiving system and the compact triple-band receiving (CTR) system^44^ are now being globally introduced to existing VLBI radio telescopes within the European VLBI Network (EVN) and the Global mm-VLBI Array (GMVA), whose observing frequencies range from 18–116 GHz. These provide new opportunities for ultra-precise astrometry and geodesy in mm-VLBI by calibrating two major sources of errors: atmospheric phase fluctuation errors, as widely demonstrated in the previous works^45,46^, and instrumental phase errors, as demonstrated in this study.

It is worth noting that since the photocurrent pulse contains numerous frequency components, multiple microwave signals at different frequencies, multiples of the effective repetition rate, can be extracted for other observation frequency bands. For instance, a 34.56 GHz microwave can be extracted and used as an LO signal for the second observation frequency band of the KVN system. Consequently, in conjunction with the simultaneous multi-frequency receiving system, our demonstrated system has the potential to surpass frequency thresholds for precision astrometric measurements.

While the current demonstration relied on an H-maser, the local atomic clock used in the KVN VLBI system, our OFC-based approach has the capability to coherently link optical frequencies to the microwave frequency domain. This approach can seamlessly complete the final step necessary for transferring optical atomic clock stability to the actual receivers in the VLBI system. This is particularly significant given the ongoing efforts to enhance clock references using higher stability frequency standards, such as the optical atomic clock disseminated via a global network^24,25^. Furthermore, high-frequency broadband VLBI is expected to be used for intercontinental comparisons of remote optical clocks with unprecedented accuracy because high-frequency broadband observation can reduce the source structure effect, a dominant error in the previous experiment^29^. For this purpose, it is vital to suppress other instrumental errors over a large width of the frequency band, and this can be accomplished by photonic-generated PCAL and LO signals.

## Materials and Methods

### Mode-locked fibre oscillator-based optical frequency comb (OFC) source

The mode-locked laser is constructed using an all-polarization-maintaining (PM) fibre-based nonlinear amplifying loop mirror (NALM) in a figure-of-nine configuration^47,48^. To enable self-starting, a nonreciprocal phase bias – comprising a half-wave plate, Faraday rotator, and octant-wave plate – is incorporated into the linear arm. The cavity length is adjusted to set the laser’s repetition rate at 40 MHz, which is the RF-comb spacing for the PCAL signal generation, and a PZT actuator attached to the mirror in the linear arm allows for modulation of this repetition rate. Note that the carrier-envelope offset frequency (f_ceo_) of the optical frequency comb is free-running in this work. However, this does not affect the performance of our system, as we utilize only the microwave signals extracted via photodetection of the pulse train. In this case, the phase noise of the repetition rate (f_rep_), which directly corresponds to pulse timing jitter, is the dominant factor that determines the stability of the generated microwave signals. Since our system is referenced to an H-maser, stabilizing f_rep_ alone is sufficient to transfer its stability to the remote microwave signal.

### H-maser-comb synchronization

The phase difference between the OFC and the OCXO (which is phase-locked to the H-maser reference) is measured by an EOS-TD. This error signal is fed into a PI servo controller, amplified by a high-gain amplifier, and then applied to the PZT actuator in the comb source to maintain synchronization. To assess the synchronization performance, an out-of-loop EOS-TD is employed (see Fig. S1 in Supplementary Information). Optical pulses from the comb are split into two paths via an optical fibre coupler, and the OCXO RF-signal is similarly divided using an RF power splitter. One set of optical and RF signals is directed to the in-loop EOS-TD for synchronization, while the other is routed to the out-of-loop EOS-TD for residual phase noise measurement. Phase noise spectra are obtained using a fast Fourier transform (FFT) analyser (Stanford Research Systems, SR770) for 1 Hz–100 kHz range and an RF spectrum analyser (Agilent, E4411B) for 100 kHz–1 MHz range.

### Fibre link stabilization

Figure S4 in Supplementary Information shows the schematic of the fibre link stabilization system. Optical pulses from the mode-locked fibre laser comb are split into two paths. One path is used as the optical reference input to the EOS-TD, passing through a dispersion-compensating fibre (DCF) and an Erbium-doped fibre amplifier (EDFA) to maximize EOS-TD detection sensitivity. The other path passes through a DCF to compensate for dispersion in the fibre link, then through an EDFA, a circulator, and a variable delay, which acts as an actuator for fibre length compensation, before being transmitted via the fibre link to the antenna. The variable delay consists of a fibre stretcher for fast delay correction and a motorized fibre delay line for compensating slower drift in the link. The reflected pulses from the end of the fibre link are photodetected by a 12-GHz p-i-n photodiode. For the RF input to the EOS-TD, two different electronic signals can be used; one is photocurrent pulses and the other is the single-frequency (1.4 GHz = 35 × 40 MHz) microwave signal (see Fig. S4b in Supplementary Information). The reference optical pulses are adjusted to be located at the middle of the rising edge of photocurrent pulses, which have an attosecond-level relative jitter^23^, in the first case and the zero-crossing of the microwave signal in the second case, as shown in Fig. S4b. The error signal from the EOS-TD is then fed back to the variable delay through a proportional-integral (PI) controller and a high-gain voltage amplifier for timing stabilization of the fibre link. To assess the link stabilization performance, an out-of-loop EOS-TD is used (see Fig. S4a). Similar to the evaluation of the H-maser-comb synchronization system, reference optical pulses are split into two paths by an optical coupler, while electronic signals are divided by a power splitter. These optical and electronic signals are directed to in-loop EOS-TD for link stabilization and to out-of-loop EOS-TD for residual noise measurement^49^.

### RF-comb signal generation

Through the timing-stabilized fibre link, optical pulses arriving at the antenna receiver room are converted to photocurrent pulses through a high-linearity photodiode (see Fig. S3a in Supplementary Information). Low-jitter photocurrent pulses are equivalent to the broadband RF comb in the frequency domain. Thereby, it can serve as a PCAL signal for the VLBI system. A low optical power of 300 μW is applied to prevent disturbance in signals from astronomical radio sources, but to extract the PCAL signal with a sufficient signal-to-noise ratio. This low incident power would be beneficial to avoid photodiode saturation.

### RF-LO generation

To achieve higher microwave signal power and to alleviate saturation effect for the LO, a fibre-based optical pulse repetition rate multiplier^39,40^ is implemented. Five-stage cascaded multiplier gives an effective repetition rate of 1.28 GHz (=40 MHz × 2^5^) (see Fig. S3b in Supplementary Information). This results in 21-dB power enhancement at 16.64 GHz (13th harmonic of 1.28 GHz), when compared to the 416th harmonic of the fundamental repetition rate of 40 MHz. Rate-multiplied optical pulses are applied to a high-speed photodiode (Freedom Photonics, FP1015C) at a bias voltage of 8 V and photocurrent of 1 mA. Then, the 13th harmonic is selected through bandpass filtering followed by an RF amplifier to match the power requirement of the receiver. In addition to the 13th harmonic, the 15th harmonic (19.2 GHz) was also obtained by changing the bandpass filter centre frequency to show multiple frequency generation capability. For the operation of KVN-Yonsei, a 16.64-GHz LO signal was injected to the VLBI receiver.

### PCAL signal extraction

The correlator combines the basebands of all telescopes to computationally form time-averaged interferometric data - measurements of the spatial coherence of the astronomical source as seen by telescope pairs, related to the Fourier-transformed image of the source. Apart of the interferometric data, the DiFX correlator can also output calibration data recovered from signals in the baseband data (e.g., switched-noise power, PCAL tones) and export them in a format readable by most VLBI postprocessing software. The PCAL tone extractor in DiFX is designed for standard geodetic and astronomical PCAL systems. With our photonic PCAL system, we were able to achieve a VLBI-friendly tone comb setup having a spacing of 40 MHz, combined with a normal VLBI receiver and the digitized baseband recordings that had 2-bit quantization.

## Supplementary information

Supplementary Information for “Optical frequency comb integration in radio telescopes: advancing signal generation and phase calibration”

## Data Availability

The data that support the findings of this study are available from the corresponding author upon request.
