# Supplementary material for: Optical frequency comb integration in radio telescopes: advancing signal generation and phase calibration
Source: Light Sci Appl. 2026 Jan 4;15:53. doi: 10.1038/s41377-025-02056-w (PMC12764496; doi:10.1038/s41377-025-02056-w)
Supplement: Supplementary file 1 — Supplementary Information for “Optical frequency comb integration in radio telescopes: advancing signal generation and phase calibration” [file 41377_2025_2056_MOESM1_ESM.pdf]

Supplementary Information for

"Optical frequency comb integration in radio telescopes: advancing signal generation and phase calibration"

Minji Hyun,[1,6] Changmin Ahn,[1,6] Junyong Choi,[1] Jihoon Baek,[1] Woosong Jeong,[1] Do-Heung Je,[2] Do-Young Byun,[2,3] Jan Wagner,[4] Myoung-Sun Heo,[5] Taehyun Jung[2] & Jungwon Kim[1,*]

[1] *Korea Advanced Institute of Science and Technology (KAIST), Daejeon 34141, Korea*

[2] *Korea Astronomy and Space Science Institute (KASI), Daejeon 34055, Korea*

[3] *University of Science and Technology (UST), Daejeon 34113, Korea*

[4]*Max-Planck Institute for Radio Astronomy, Bonn 53121, Germany*

[5]*Korea Research Institute of Standards and Science (KRISS), Daejeon 34113, Korea*

[6]*These authors contributed equally: Minji Hyun and Changmin Ahn*

**e-mail: jungwon.kim@kaist.ac.kr*

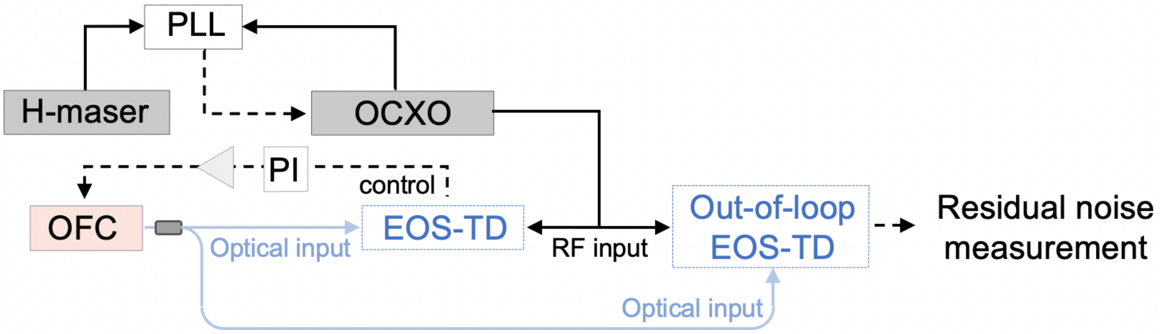

Figure S1 | Schematic of H-maser-comb synchronization. EOS-TD, electro-optic sampling-based timing detector; OCXO, oven controlled crystal oscillator; OFC, optical frequency comb (mode-locked Er-fibre laser comb source); PI, proportional-integral servo controller; PLL, phase-locked loop.

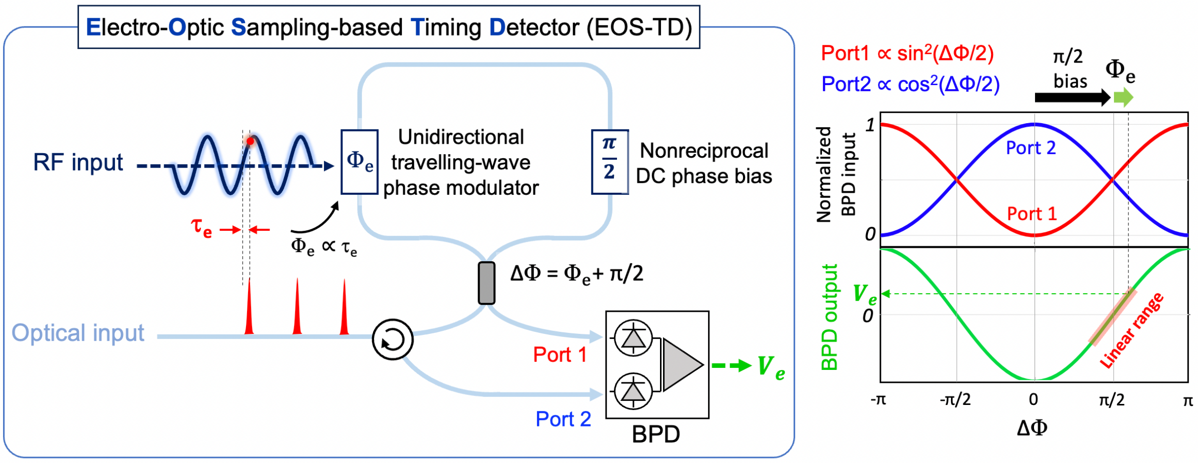

Figure S2 | The internal structure and operation principle of the EOS-TD. BPD, balanced photodetector.

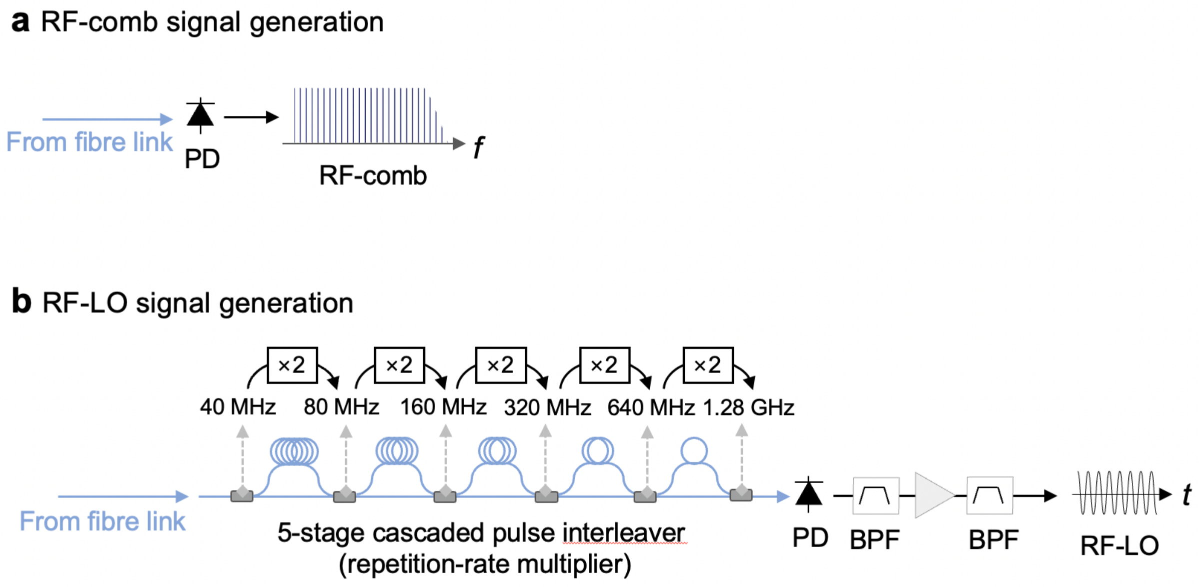

Figure S3 | Schematic of **a,** RF-comb signal generation and **b,** RF-LO signal generation from the fibre link-delivered optical pulse train at the antenna receiver room. BPF, RF bandpass filter; PD, photodiode with 50-ohm termination.

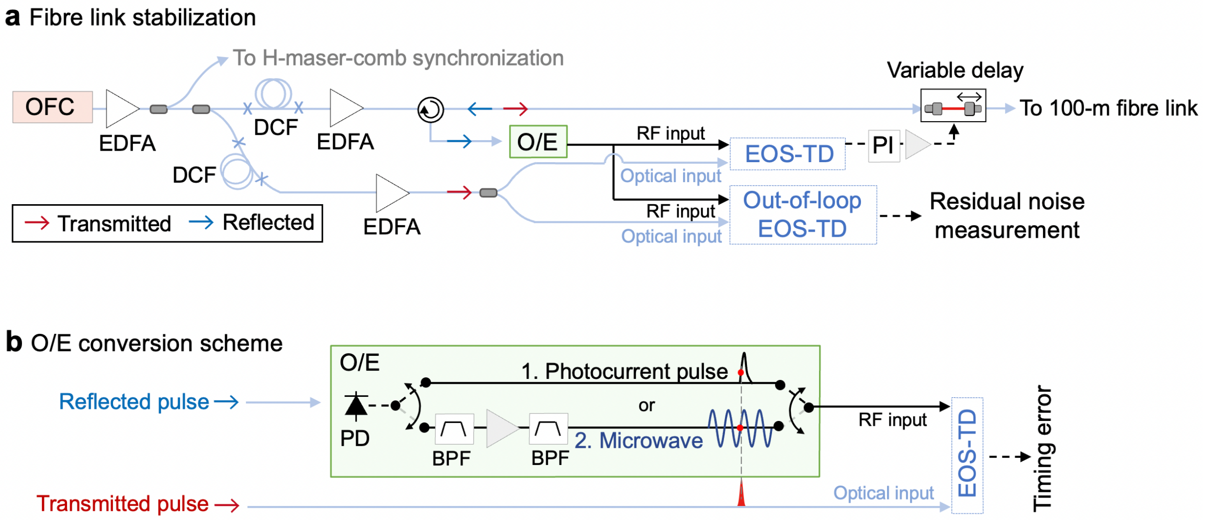

Figure S4 | Schematic of **a,** Fibre link stabilization system and **b,** O/E conversion scheme. BPF, RF bandpass filter; DCF, dispersion compensating fibre; EDFA, Erbium-doped fibre amplifier; EOS-TD, electro-optic sampling-based timing detector; O/E, optical-to-electrical conversion; OFC, optical frequency comb source (40-MHz mode-locked Er-fibre laser comb); PD, photodiode with 50-ohm termination; PI, proportional-integral servo controller.

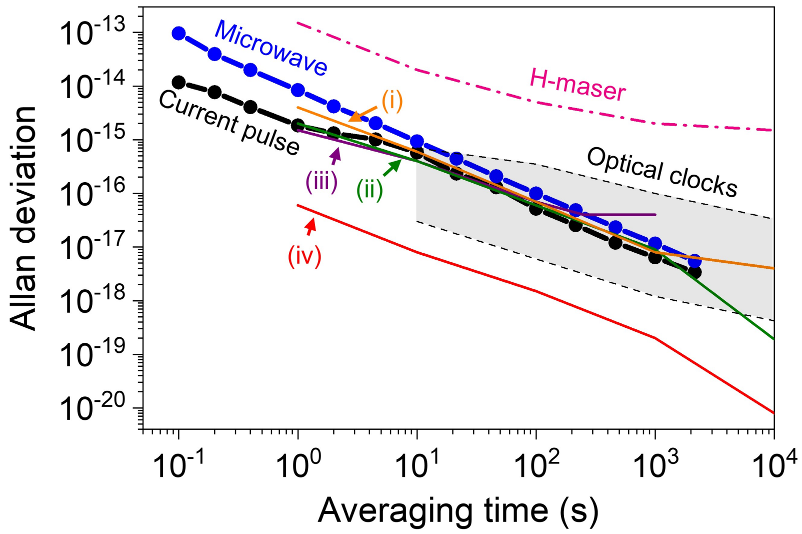

Figure S5 | Comparison of the stability of optical frequency comb transfer over fibre links. Optical phase detection-based method (curve (i), ref. S1), EOS-TD-based methods (curve (ii), ref. S2 and curve (iii), ref. S3) and optical cross-correlator-based method (curve (iv), ref. S4).

References

S1. Marra, G., Margolis, H. S. & Richardson, D. J. Dissemination of an optical frequency comb over fiber with 3×10^{-18} fractional accuracy. *Opt. Express* **20**, 1775-1782 (2012).

S2. Ning, B. et al. High-precision distribution of highly stable optical pulse trans with 8.8×10^{-19} instability. *Sci. Rep.* **4**, 5109 (2014).

S3. Jung, K. et al. Frequency comb-based microwave transfer over fiber with 7×10^{-19} instability using fiber-loop optical-microwave phase detectors. *Opt. Lett.* **39**, 1577-1580 (2014).

S4. Xin, M. et al. Attosecond precision multi-kilometer laser-microwave network. *Light Sci. Appl.* **6**, e16187 (2017).
